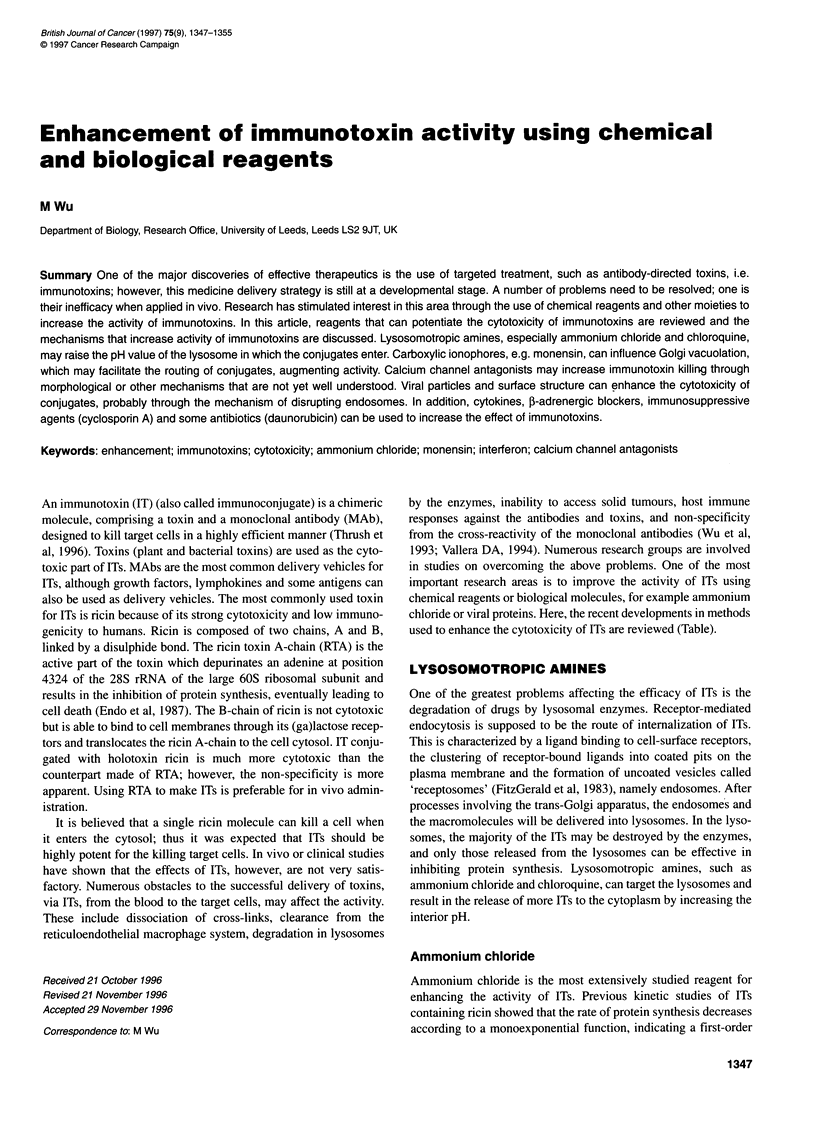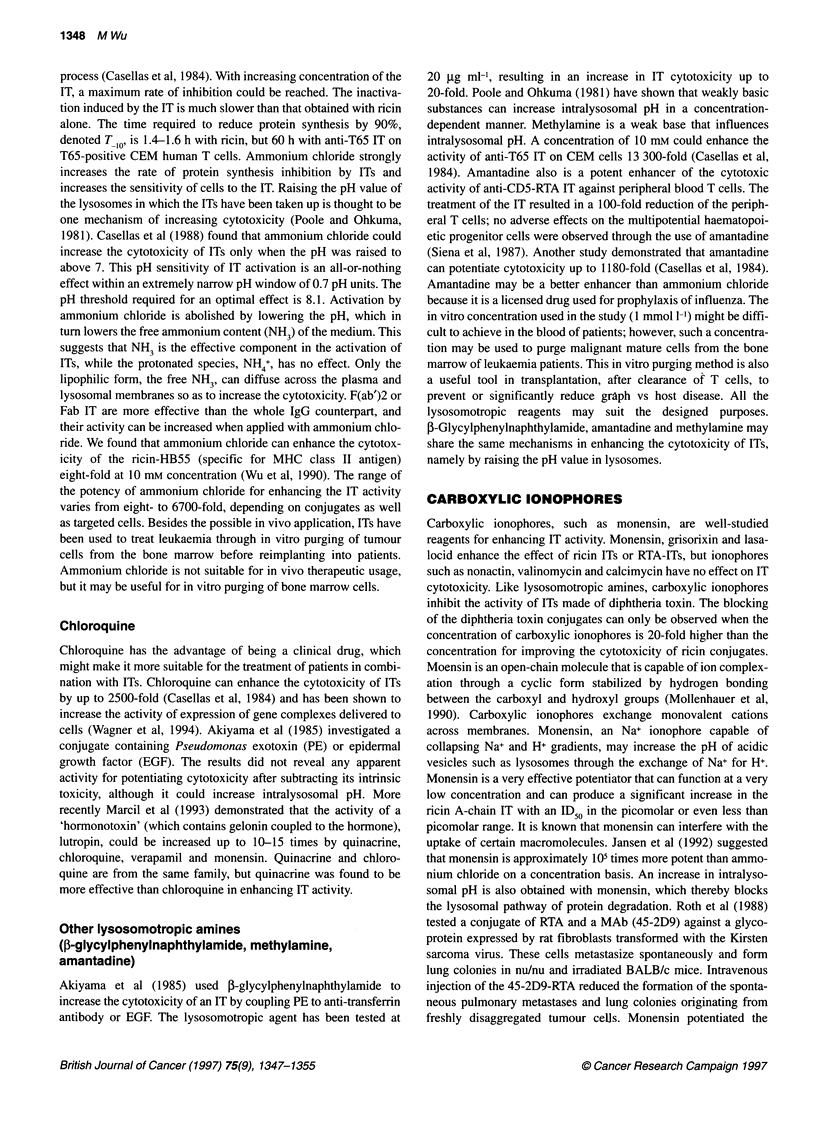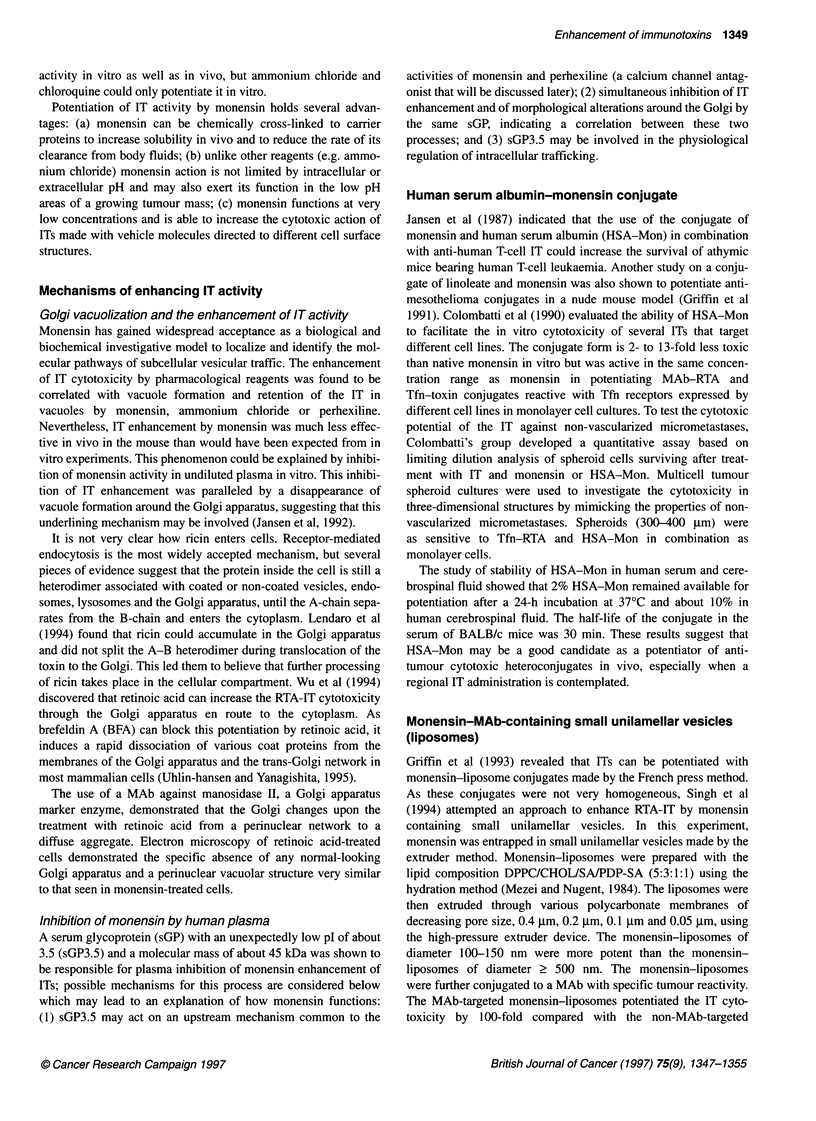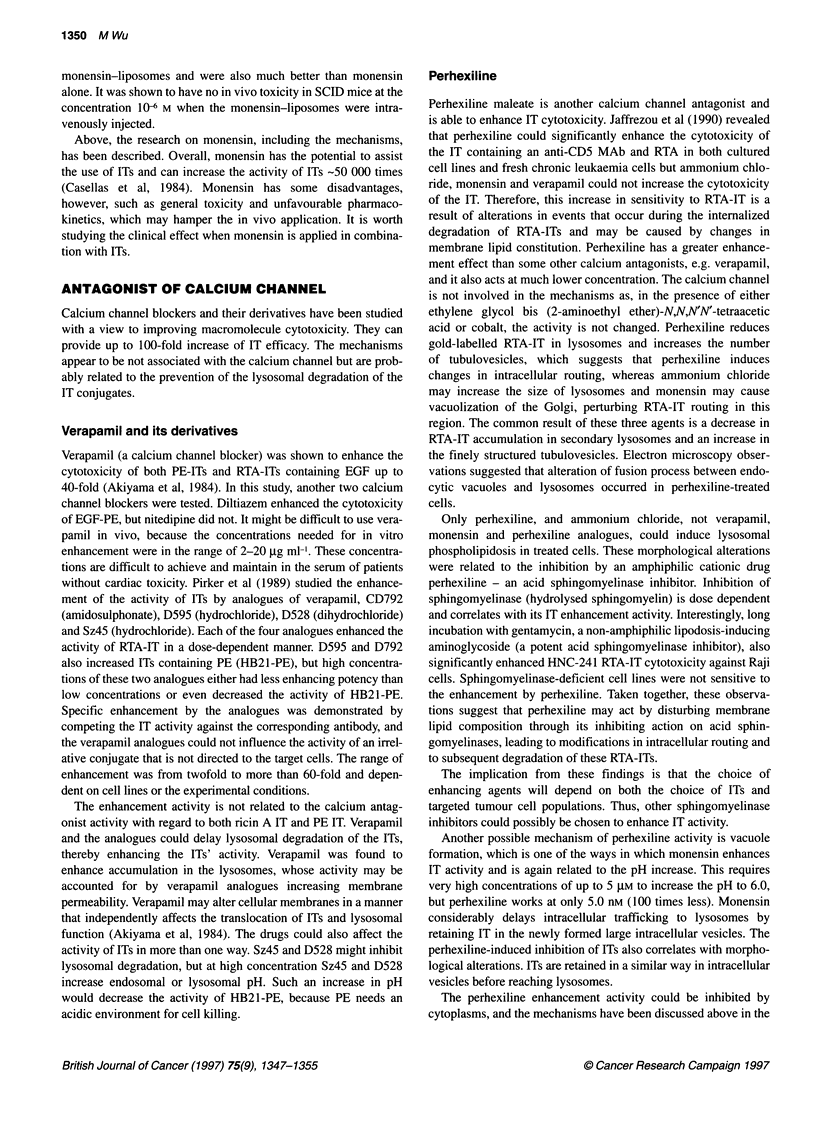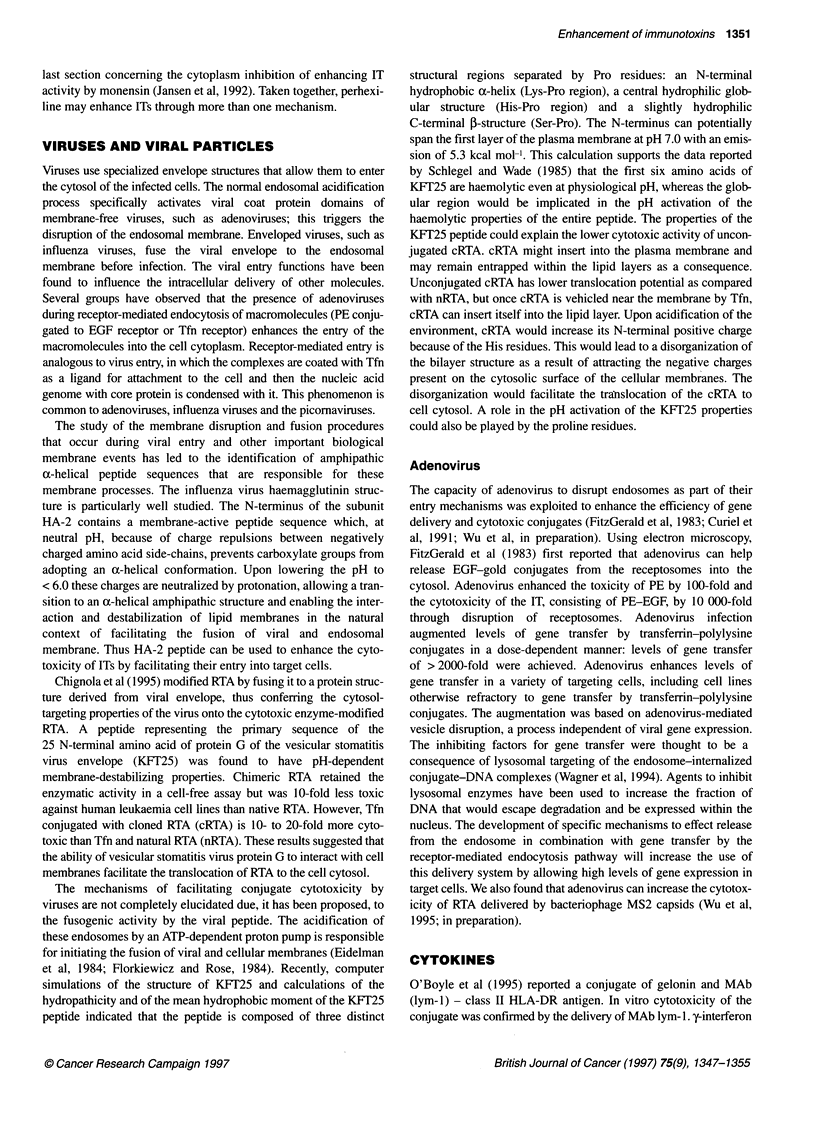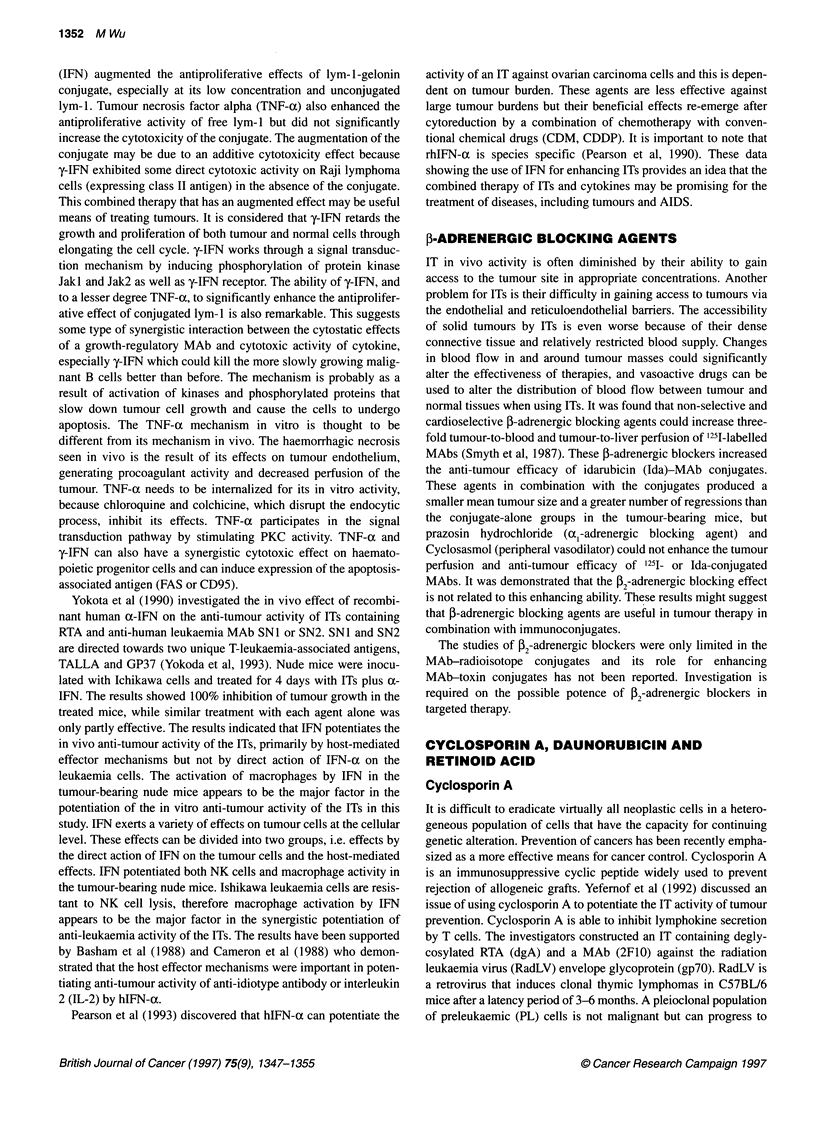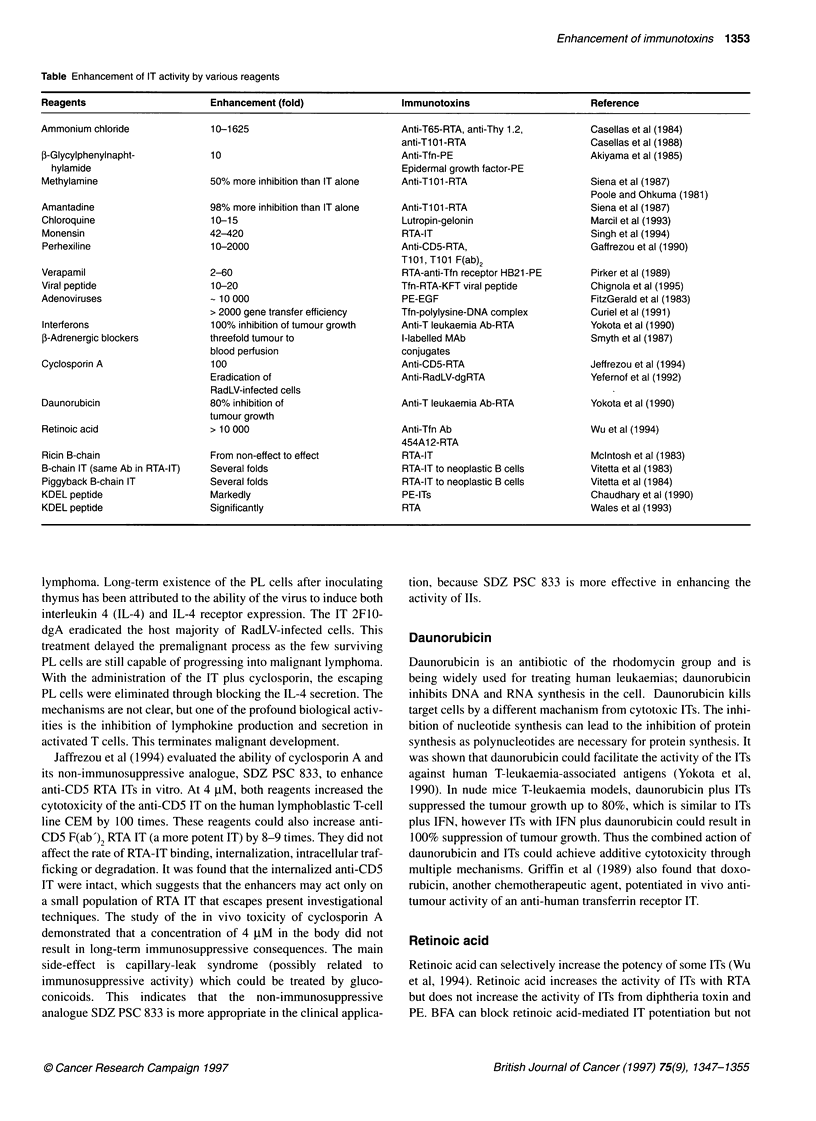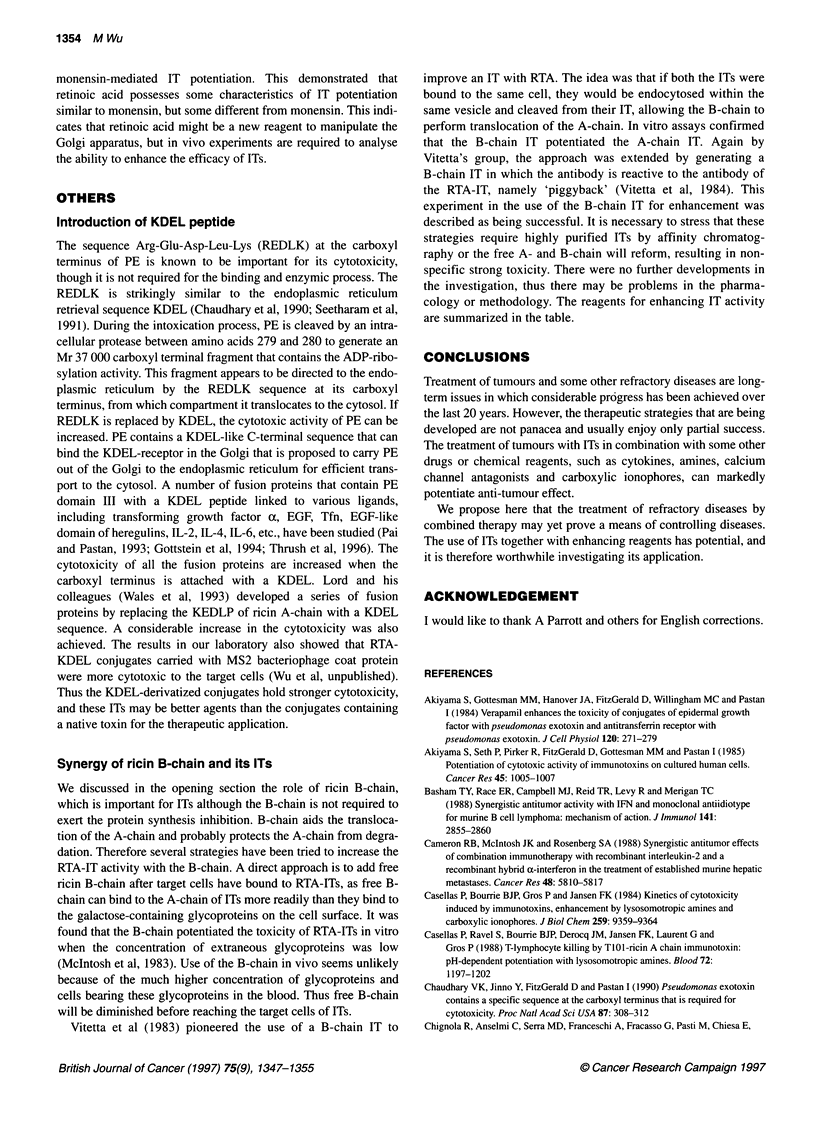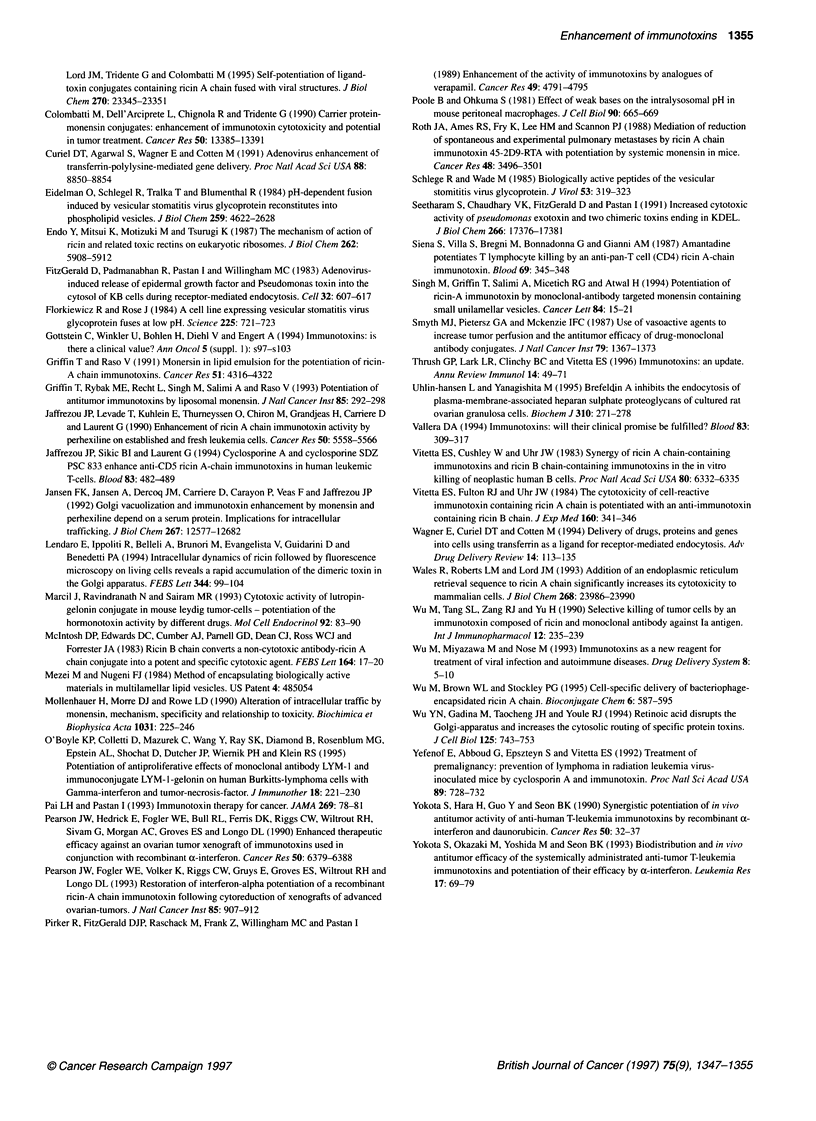# Enhancement of immunotoxin activity using chemical and biological reagents.

**DOI:** 10.1038/bjc.1997.228

**Published:** 1997

**Authors:** M. Wu

**Affiliations:** Department of Biology, University of Leeds, UK.

## Abstract

One of the major discoveries of effective therapeutics is the use of targeted treatment, such as antibody-directed toxins, i.e. immunotoxins; however, this medicine delivery strategy is still at a developmental stage. A number of problems need to be resolved; one is their inefficacy when applied in vivo. Research has stimulated interest in this area through the use of chemical reagents and other moieties to increase the activity of immunotoxins. In this article, reagents that can potentiate the cytotoxicity of immunotoxins are reviewed and the mechanisms that increase activity of immunotoxins are discussed. Lysosomotropic amines, especially ammonium chloride and chloroquine, may raise the pH value of the lysosome in which the conjugates enter. Carboxylic ionophores, e.g. monensin, can influence Golgi vacuolation, which may facilitate the routing of conjugates, augmenting activity. Calcium channel antagonists may increase immunotoxin killing through morphological or other mechanisms that are not yet well understood. Viral particles and surface structure can enhance the cytotoxicity of conjugates, probably through the mechanism of disrupting endosomes. In addition, cytokines, beta-adrenergic blockers, immunosuppressive agents (cyclosporin A) and some antibiotics (daunorubicin) can be used to increase the effect of immunotoxins.


British Journal of Cancer (1997) 75(9), 1347–1355
© 1997 Cancer Research Campaign

### Enhancement of immunotoxin activity using chemical and biological reagents

**M Wu**

Department of Biology, Research Office, University of Leeds, Leeds LS2 9JT, UK

**Summary** One of the major discoveries of effective therapeutics is the use of targeted treatment, such as antibody-directed toxins, i.e. immunotoxins; however, this medicine delivery strategy is still at a developmental stage. A number of problems need to be resolved; one is their inefficacy when applied in vivo. Research has stimulated interest in this area through the use of chemical reagents and other moieties to increase the activity of immunotoxins. In this article, reagents that can potentiate the cytotoxicity of immunotoxins are reviewed and the mechanisms that increase activity of immunotoxins are discussed. Lysosomotropic amines, especially ammonium chloride and chloroquine, may raise the pH value of the lysosome in which the conjugates enter. Carboxylic ionophores, e.g. monensin, can influence Golgi vacuolation, which may facilitate the routing of conjugates, augmenting activity. Calcium channel antagonists may increase immunotoxin killing through morphological or other mechanisms that are not yet well understood. Viral particles and surface structure can enhance the cytotoxicity of conjugates, probably through the mechanism of disrupting endosomes. In addition, cytokines, β-adrenergic blockers, immunosuppressive agents (cyclosporin A) and some antibiotics (daunorubicin) can be used to increase the effect of immunotoxins.

**Keywords**: enhancement; immunotoxins; cytotoxicity; ammonium chloride; monensin; interferon; calcium channel antagonists

An immunotoxin (IT) (also called immunoconjugate) is a chimeric molecule, comprising a toxin and a monoclonal antibody (MAb), designed to kill target cells in a highly efficient manner (Thrush et al, 1996). Toxins (plant and bacterial toxins) are used as the cytotoxic part of ITs. MAbs are the most common delivery vehicles for ITs, although growth factors, lymphokines and some antigens can also be used as delivery vehicles. The most commonly used toxin for ITs is ricin because of its strong cytotoxicity and low immunogenicity to humans. Ricin is composed of two chains, A and B, linked by a disulphide bond. The ricin toxin A-chain (RTA) is the active part of the toxin which depurinates an adenine at position 4324 of the 28S rRNA of the large 60S ribosomal subunit and results in the inhibition of protein synthesis, eventually leading to cell death (Endo et al, 1987). The B-chain of ricin is not cytotoxic but is able to bind to cell membranes through its (ga)lactose receptors and translocates the ricin A-chain to the cell cytosol. IT conjugated with holotoxin ricin is much more cytotoxic than the counterpart made of RTA; however, the non-specificity is more apparent. Using RTA to make ITs is preferable for in vivo administration.

It is believed that a single ricin molecule can kill a cell when it enters the cytosol; thus it was expected that ITs should be highly potent for the killing target cells. In vivo or clinical studies have shown that the effects of ITs, however, are not very satisfactory. Numerous obstacles to the successful delivery of toxins, via ITs, from the blood to the target cells, may affect the activity. These include dissociation of cross-links, clearance from the reticuloendothelial macrophage system, degradation in lysosomes by the enzymes, inability to access solid tumours, host immune responses against the antibodies and toxins, and non-specificity from the cross-reactivity of the monoclonal antibodies (Wu et al, 1993; Vallera DA, 1994). Numerous research groups are involved in studies on overcoming the above problems. One of the most important research areas is to improve the activity of ITs using chemical reagents or biological molecules, for example ammonium chloride or viral proteins. Here, the recent developments in methods used to enhance the cytotoxicity of ITs are reviewed (Table).

*Received 21 October 1996*
*Revised 21 November 1996*
*Accepted 29 November 1996*

*Correspondence to*: M Wu

### LYSOSOMOTROPIC AMINES

One of the greatest problems affecting the efficacy of ITs is the degradation of drugs by lysosomal enzymes. Receptor-mediated endocytosis is supposed to be the route of internalization of ITs. This is characterized by a ligand binding to cell-surface receptors, the clustering of receptor-bound ligands into coated pits on the plasma membrane and the formation of uncoated vesicles called ‘receptosomes’ (FitzGerald et al, 1983), namely endosomes. After processes involving the trans-Golgi apparatus, the endosomes and the macromolecules will be delivered into lysosomes. In the lysosomes, the majority of the ITs may be destroyed by the enzymes, and only those released from the lysosomes can be effective in inhibiting protein synthesis. Lysosomotropic amines, such as ammonium chloride and chloroquine, can target the lysosomes and result in the release of more ITs to the cytoplasm by increasing the interior pH.

### Ammonium chloride

Ammonium chloride is the most extensively studied reagent for enhancing the activity of ITs. Previous kinetic studies of ITs containing ricin showed that the rate of protein synthesis decreases according to a monoexponential function, indicating a first-order process (Casellas et al, 1984). With increasing concentration of the IT, a maximum rate of inhibition could be reached. The inactivation induced by the IT is much slower than that obtained with ricin alone. The time required to reduce protein synthesis by 90%, denoted $T_{-10}$, is 1.4–1.6 h with ricin, but 60 h with anti-T65 IT on T65-positive CEM human T cells. Ammonium chloride strongly increases the rate of protein synthesis inhibition by ITs and increases the sensitivity of cells to the IT. Raising the pH value of the lysosomes in which the ITs have been taken up is thought to be one mechanism of increasing cytotoxicity (Poole and Ohkuma, 1981). Casellas et al (1988) found that ammonium chloride could increase the cytotoxicity of ITs only when the pH was raised to above 7. This pH sensitivity of IT activation is an all-or-nothing effect within an extremely narrow pH window of 0.7 pH units. The pH threshold required for an optimal effect is 8.1. Activation by ammonium chloride is abolished by lowering the pH, which in turn lowers the free ammonium content ($NH_3$) of the medium. This suggests that $NH_3$ is the effective component in the activation of ITs, while the protonated species, $NH_4^+$, has no effect. Only the lipophilic form, the free $NH_3$, can diffuse across the plasma and lysosomal membranes so as to increase the cytotoxicity. F(ab′)2 or Fab IT are more effective than the whole IgG counterpart, and their activity can be increased when applied with ammonium chloride. We found that ammonium chloride can enhance the cytotoxicity of the ricin-HB55 (specific for MHC class II antigen) eight-fold at 10 mM concentration (Wu et al, 1990). The range of the potency of ammonium chloride for enhancing the IT activity varies from eight- to 6700-fold, depending on conjugates as well as targeted cells. Besides the possible in vivo application, ITs have been used to treat leukaemia through in vitro purging of tumour cells from the bone marrow before reimplanting into patients. Ammonium chloride is not suitable for in vivo therapeutic usage, but it may be useful for in vitro purging of bone marrow cells.

### Chloroquine

Chloroquine has the advantage of being a clinical drug, which might make it more suitable for the treatment of patients in combination with ITs. Chloroquine can enhance the cytotoxicity of ITs by up to 2500-fold (Casellas et al, 1984) and has been shown to increase the activity of expression of gene complexes delivered to cells (Wagner et al, 1994). Akiyama et al (1985) investigated a conjugate containing *Pseudomonas* exotoxin (PE) or epidermal growth factor (EGF). The results did not reveal any apparent activity for potentiating cytotoxicity after subtracting its intrinsic toxicity, although it could increase intralysosomal pH. More recently Marcil et al (1993) demonstrated that the activity of a ‘hormonotoxin’ (which contains gelonin coupled to the hormone), lutropin, could be increased up to 10–15 times by quinacrine, chloroquine, verapamil and monensin. Quinacrine and chloroquine are from the same family, but quinacrine was found to be more effective than chloroquine in enhancing IT activity.

### Other lysosomotropic amines (β-glycylphenylnaphthylamide, methylamine, amantadine)

Akiyama et al (1985) used β-glycylphenylnaphthylamide to increase the cytotoxicity of an IT by coupling PE to anti-transferrin antibody or EGF. The lysosomotropic agent has been tested at 20 μg ml$^{-1}$, resulting in an increase in IT cytotoxicity up to 20-fold. Poole and Ohkuma (1981) have shown that weakly basic substances can increase intralysosomal pH in a concentration-dependent manner. Methylamine is a weak base that influences intralysosomal pH. A concentration of 10 mM could enhance the activity of anti-T65 IT on CEM cells 13 300-fold (Casellas et al, 1984). Amantadine also is a potent enhancer of the cytotoxic activity of anti-CD5-RTA IT against peripheral blood T cells. The treatment of the IT resulted in a 100-fold reduction of the peripheral T cells; no adverse effects on the multipotential haematopoietic progenitor cells were observed through the use of amantadine (Siena et al, 1987). Another study demonstrated that amantadine can potentiate cytotoxicity up to 1180-fold (Casellas et al, 1984). Amantadine may be a better enhancer than ammonium chloride because it is a licensed drug used for prophylaxis of influenza. The in vitro concentration used in the study (1 mmol l$^{-1}$) might be difficult to achieve in the blood of patients; however, such a concentration may be used to purge malignant mature cells from the bone marrow of leukaemia patients. This in vitro purging method is also a useful tool in transplantation, after clearance of T cells, to prevent or significantly reduce gràph vs host disease. All the lysosomotropic reagents may suit the designed purposes. β-Glycylphenylnaphthylamide, amantadine and methylamine may share the same mechanisms in enhancing the cytotoxicity of ITs, namely by raising the pH value in lysosomes.

### CARBOXYLIC IONOPHORES

Carboxylic ionophores, such as monensin, are well-studied reagents for enhancing IT activity. Monensin, grisorixin and lasalocid enhance the effect of ricin ITs or RTA-ITs, but ionophores such as nonactin, valinomycin and calcimycin have no effect on IT cytotoxicity. Like lysosomotropic amines, carboxylic ionophores inhibit the activity of ITs made of diphtheria toxin. The blocking of the diphtheria toxin conjugates can only be observed when the concentration of carboxylic ionophores is 20-fold higher than the concentration for improving the cytotoxicity of ricin conjugates. Moensin is an open-chain molecule that is capable of ion complexation through a cyclic form stabilized by hydrogen bonding between the carboxyl and hydroxyl groups (Mollenhauer et al, 1990). Carboxylic ionophores exchange monovalent cations across membranes. Monensin, an $Na^+$ ionophore capable of collapsing $Na^+$ and $H^+$ gradients, may increase the pH of acidic vesicles such as lysosomes through the exchange of $Na^+$ for $H^+$. Monensin is a very effective potentiator that can function at a very low concentration and can produce a significant increase in the ricin A-chain IT with an $ID_{50}$ in the picomolar or even less than picomolar range. It is known that monensin can interfere with the uptake of certain macromolecules. Jansen et al (1992) suggested that monensin is approximately $10^5$ times more potent than ammonium chloride on a concentration basis. An increase in intralysosomal pH is also obtained with monensin, which thereby blocks the lysosomal pathway of protein degradation. Roth et al (1988) tested a conjugate of RTA and a MAb (45-2D9) against a glycoprotein expressed by rat fibroblasts transformed with the Kirsten sarcoma virus. These cells metastasize spontaneously and form lung colonies in nu/nu and irradiated BALB/c mice. Intravenous injection of the 45-2D9-RTA reduced the formation of the spontaneous pulmonary metastases and lung colonies originating from freshly disaggregated tumour cells. Monensin potentiated the

© Cancer Research Campaign 1997

activity in vitro as well as in vivo, but ammonium chloride and chloroquine could only potentiate it in vitro.

Potentiation of IT activity by monensin holds several advantages: (a) monensin can be chemically cross-linked to carrier proteins to increase solubility in vivo and to reduce the rate of its clearance from body fluids; (b) unlike other reagents (e.g. ammonium chloride) monensin action is not limited by intracellular or extracellular pH and may also exert its function in the low pH areas of a growing tumour mass; (c) monensin functions at very low concentrations and is able to increase the cytotoxic action of ITs made with vehicle molecules directed to different cell surface structures.

### Mechanisms of enhancing IT activity

### *Golgi vacuolization and the enhancement of IT activity*

Monensin has gained widespread acceptance as a biological and biochemical investigative model to localize and identify the molecular pathways of subcellular vesicular traffic. The enhancement of IT cytotoxicity by pharmacological reagents was found to be correlated with vacuole formation and retention of the IT in vacuoles by monensin, ammonium chloride or perhexiline. Nevertheless, IT enhancement by monensin was much less effective in vivo in the mouse than would have been expected from in vitro experiments. This phenomenon could be explained by inhibition of monensin activity in undiluted plasma in vitro. This inhibition of IT enhancement was paralleled by a disappearance of vacuole formation around the Golgi apparatus, suggesting that this underlining mechanism may be involved (Jansen et al, 1992).

It is not very clear how ricin enters cells. Receptor-mediated endocytosis is the most widely accepted mechanism, but several pieces of evidence suggest that the protein inside the cell is still a heterodimer associated with coated or non-coated vesicles, endosomes, lysosomes and the Golgi apparatus, until the A-chain separates from the B-chain and enters the cytoplasm. Lendaro et al (1994) found that ricin could accumulate in the Golgi apparatus and did not split the A–B heterodimer during translocation of the toxin to the Golgi. This led them to believe that further processing of ricin takes place in the cellular compartment. Wu et al (1994) discovered that retinoic acid can increase the RTA-IT cytotoxicity through the Golgi apparatus en route to the cytoplasm. As brefeldin A (BFA) can block this potentiation by retinoic acid, it induces a rapid dissociation of various coat proteins from the membranes of the Golgi apparatus and the trans-Golgi network in most mammalian cells (Uhlin-hansen and Yanagishita, 1995).

The use of a MAb against manosidase II, a Golgi apparatus marker enzyme, demonstrated that the Golgi changes upon the treatment with retinoic acid from a perinuclear network to a diffuse aggregate. Electron microscopy of retinoic acid-treated cells demonstrated the specific absence of any normal-looking Golgi apparatus and a perinuclear vacuolar structure very similar to that seen in monensin-treated cells.

### *Inhibition of monensin by human plasma*

A serum glycoprotein (sGP) with an unexpectedly low pI of about 3.5 (sGP3.5) and a molecular mass of about 45 kDa was shown to be responsible for plasma inhibition of monensin enhancement of ITs; possible mechanisms for this process are considered below which may lead to an explanation of how monensin functions: (1) sGP3.5 may act on an upstream mechanism common to the activities of monensin and perhexiline (a calcium channel antagonist that will be discussed later); (2) simultaneous inhibition of IT enhancement and of morphological alterations around the Golgi by the same sGP, indicating a correlation between these two processes; and (3) sGP3.5 may be involved in the physiological regulation of intracellular trafficking.

### Human serum albumin–monensin conjugate

Jansen et al (1987) indicated that the use of the conjugate of monensin and human serum albumin (HSA–Mon) in combination with anti-human T-cell IT could increase the survival of athymic mice bearing human T-cell leukaemia. Another study on a conjugate of linoleate and monensin was also shown to potentiate anti-mesothelioma conjugates in a nude mouse model (Griffin et al 1991). Colombatti et al (1990) evaluated the ability of HSA–Mon to facilitate the in vitro cytotoxicity of several ITs that target different cell lines. The conjugate form is 2- to 13-fold less toxic than native monensin in vitro but was active in the same concentration range as monensin in potentiating MAb–RTA and Tfn–toxin conjugates reactive with Tfn receptors expressed by different cell lines in monolayer cell cultures. To test the cytotoxic potential of the IT against non-vascularized micrometastases, Colombatti's group developed a quantitative assay based on limiting dilution analysis of spheroid cells surviving after treatment with IT and monensin or HSA–Mon. Multicell tumour spheroid cultures were used to investigate the cytotoxicity in three-dimensional structures by mimicking the properties of non-vascularized micrometastases. Spheroids (300–400 μm) were as sensitive to Tfn–RTA and HSA–Mon in combination as monolayer cells.

The study of stability of HSA–Mon in human serum and cerebrospinal fluid showed that 2% HSA–Mon remained available for potentiation after a 24-h incubation at 37°C and about 10% in human cerebrospinal fluid. The half-life of the conjugate in the serum of BALB/c mice was 30 min. These results suggest that HSA–Mon may be a good candidate as a potentiator of anti-tumour cytotoxic heteroconjugates in vivo, especially when a regional IT administration is contemplated.

### Monensin–MAb-containing small unilamellar vesicles (liposomes)

Griffin et al (1993) revealed that ITs can be potentiated with monensin–liposome conjugates made by the French press method. As these conjugates were not very homogeneous, Singh et al (1994) attempted an approach to enhance RTA-IT by monensin containing small unilamellar vesicles. In this experiment, monensin was entrapped in small unilamellar vesicles made by the extruder method. Monensin–liposomes were prepared with the lipid composition DPPC/CHOL/SA/PDP-SA (5:3:1:1) using the hydration method (Mezei and Nugent, 1984). The liposomes were then extruded through various polycarbonate membranes of decreasing pore size, 0.4 μm, 0.2 μm, 0.1 μm and 0.05 μm, using the high-pressure extruder device. The monensin–liposomes of diameter 100–150 nm were more potent than the monensin–liposomes of diameter ≥ 500 nm. The monensin–liposomes were further conjugated to a MAb with specific tumour reactivity. The MAb-targeted monensin–liposomes potentiated the IT cytotoxicity by 100-fold compared with the non-MAb-targeted

© Cancer Research Campaign 1997

monensin–liposomes and were also much better than monensin alone. It was shown to have no in vivo toxicity in SCID mice at the concentration $10^{-6}$ M when the monensin–liposomes were intravenously injected.

Above, the research on monensin, including the mechanisms, has been described. Overall, monensin has the potential to assist the use of ITs and can increase the activity of ITs ~50 000 times (Casellas et al, 1984). Monensin has some disadvantages, however, such as general toxicity and unfavourable pharmacokinetics, which may hamper the in vivo application. It is worth studying the clinical effect when monensin is applied in combination with ITs.

### ANTAGONIST OF CALCIUM CHANNEL

Calcium channel blockers and their derivatives have been studied with a view to improving macromolecule cytotoxicity. They can provide up to 100-fold increase of IT efficacy. The mechanisms appear to be not associated with the calcium channel but are probably related to the prevention of the lysosomal degradation of the IT conjugates.

### Verapamil and its derivatives

Verapamil (a calcium channel blocker) was shown to enhance the cytotoxicity of both PE-ITs and RTA-ITs containing EGF up to 40-fold (Akiyama et al, 1984). In this study, another two calcium channel blockers were tested. Diltiazem enhanced the cytotoxicity of EGF-PE, but nitedipine did not. It might be difficult to use verapamil in vivo, because the concentrations needed for in vitro enhancement were in the range of 2–20 μg $ml^{-1}$. These concentrations are difficult to achieve and maintain in the serum of patients without cardiac toxicity. Pirker et al (1989) studied the enhancement of the activity of ITs by analogues of verapamil, CD792 (amidosulphonate), D595 (hydrochloride), D528 (dihydrochloride) and Sz45 (hydrochloride). Each of the four analogues enhanced the activity of RTA-IT in a dose-dependent manner. D595 and D792 also increased ITs containing PE (HB21-PE), but high concentrations of these two analogues either had less enhancing potency than low concentrations or even decreased the activity of HB21-PE. Specific enhancement by the analogues was demonstrated by competing the IT activity against the corresponding antibody, and the verapamil analogues could not influence the activity of an irrelative conjugate that is not directed to the target cells. The range of enhancement was from twofold to more than 60-fold and dependent on cell lines or the experimental conditions.

The enhancement activity is not related to the calcium antagonist activity with regard to both ricin A IT and PE IT. Verapamil and the analogues could delay lysosomal degradation of the ITs, thereby enhancing the ITs' activity. Verapamil was found to enhance accumulation in the lysosomes, whose activity may be accounted for by verapamil analogues increasing membrane permeability. Verapamil may alter cellular membranes in a manner that independently affects the translocation of ITs and lysosomal function (Akiyama et al, 1984). The drugs could also affect the activity of ITs in more than one way. Sz45 and D528 might inhibit lysosomal degradation, but at high concentration Sz45 and D528 increase endosomal or lysosomal pH. Such an increase in pH would decrease the activity of HB21-PE, because PE needs an acidic environment for cell killing.

### Perhexiline

Perhexiline maleate is another calcium channel antagonist and is able to enhance IT cytotoxicity. Jaffrezou et al (1990) revealed that perhexiline could significantly enhance the cytotoxicity of the IT containing an anti-CD5 MAb and RTA in both cultured cell lines and fresh chronic leukaemia cells but ammonium chloride, monensin and verapamil could not increase the cytotoxicity of the IT. Therefore, this increase in sensitivity to RTA-IT is a result of alterations in events that occur during the internalized degradation of RTA-ITs and may be caused by changes in membrane lipid constitution. Perhexiline has a greater enhancement effect than some other calcium antagonists, e.g. verapamil, and it also acts at much lower concentration. The calcium channel is not involved in the mechanisms as, in the presence of either ethylene glycol bis (2-aminoethyl ether)-*N,N,N′N′*-tetraacetic acid or cobalt, the activity is not changed. Perhexiline reduces gold-labelled RTA-IT in lysosomes and increases the number of tubulovesicles, which suggests that perhexiline induces changes in intracellular routing, whereas ammonium chloride may increase the size of lysosomes and monensin may cause vacuolization of the Golgi, perturbing RTA-IT routing in this region. The common result of these three agents is a decrease in RTA-IT accumulation in secondary lysosomes and an increase in the finely structured tubulovesicles. Electron microscopy observations suggested that alteration of fusion process between endocytic vacuoles and lysosomes occurred in perhexiline-treated cells.

Only perhexiline, and ammonium chloride, not verapamil, monensin and perhexiline analogues, could induce lysosomal phospholipidosis in treated cells. These morphological alterations were related to the inhibition by an amphiphilic cationic drug perhexiline – an acid sphingomyelinase inhibitor. Inhibition of sphingomyelinase (hydrolysed sphingomyelin) is dose dependent and correlates with its IT enhancement activity. Interestingly, long incubation with gentamycin, a non-amphiphilic lipodosis-inducing aminoglycoside (a potent acid sphingomyelinase inhibitor), also significantly enhanced HNC-241 RTA-IT cytotoxicity against Raji cells. Sphingomyelinase-deficient cell lines were not sensitive to the enhancement by perhexiline. Taken together, these observations suggest that perhexiline may act by disturbing membrane lipid composition through its inhibiting action on acid sphingomyelinases, leading to modifications in intracellular routing and to subsequent degradation of these RTA-ITs.

The implication from these findings is that the choice of enhancing agents will depend on both the choice of ITs and targeted tumour cell populations. Thus, other sphingomyelinase inhibitors could possibly be chosen to enhance IT activity.

Another possible mechanism of perhexiline activity is vacuole formation, which is one of the ways in which monensin enhances IT activity and is again related to the pH increase. This requires very high concentrations of up to 5 μM to increase the pH to 6.0, but perhexiline works at only 5.0 nM (100 times less). Monensin considerably delays intracellular trafficking to lysosomes by retaining IT in the newly formed large intracellular vesicles. The perhexiline-induced inhibition of ITs also correlates with morphological alterations. ITs are retained in a similar way in intracellular vesicles before reaching lysosomes.

The perhexiline enhancement activity could be inhibited by cytoplasms, and the mechanisms have been discussed above in the

 *© Cancer Research Campaign 1997*

last section concerning the cytoplasm inhibition of enhancing IT activity by monensin (Jansen et al, 1992). Taken together, perhexiline may enhance ITs through more than one mechanism.

### VIRUSES AND VIRAL PARTICLES

Viruses use specialized envelope structures that allow them to enter the cytosol of the infected cells. The normal endosomal acidification process specifically activates viral coat protein domains of membrane-free viruses, such as adenoviruses; this triggers the disruption of the endosomal membrane. Enveloped viruses, such as influenza viruses, fuse the viral envelope to the endosomal membrane before infection. The viral entry functions have been found to influence the intracellular delivery of other molecules. Several groups have observed that the presence of adenoviruses during receptor-mediated endocytosis of macromolecules (PE conjugated to EGF receptor or Tfn receptor) enhances the entry of the macromolecules into the cell cytoplasm. Receptor-mediated entry is analogous to virus entry, in which the complexes are coated with Tfn as a ligand for attachment to the cell and then the nucleic acid genome with core protein is condensed with it. This phenomenon is common to adenoviruses, influenza viruses and the picornaviruses.

The study of the membrane disruption and fusion procedures that occur during viral entry and other important biological membrane events has led to the identification of amphipathic α-helical peptide sequences that are responsible for these membrane processes. The influenza virus haemagglutinin structure is particularly well studied. The N-terminus of the subunit HA-2 contains a membrane-active peptide sequence which, at neutral pH, because of charge repulsions between negatively charged amino acid side-chains, prevents carboxylate groups from adopting an α-helical conformation. Upon lowering the pH to < 6.0 these charges are neutralized by protonation, allowing a transition to an α-helical amphipathic structure and enabling the interaction and destabilization of lipid membranes in the natural context of facilitating the fusion of viral and endosomal membrane. Thus HA-2 peptide can be used to enhance the cytotoxicity of ITs by facilitating their entry into target cells.

Chignola et al (1995) modified RTA by fusing it to a protein structure derived from viral envelope, thus conferring the cytosol-targeting properties of the virus onto the cytotoxic enzyme-modified RTA. A peptide representing the primary sequence of the 25 N-terminal amino acid of protein G of the vesicular stomatitis virus envelope (KFT25) was found to have pH-dependent membrane-destabilizing properties. Chimeric RTA retained the enzymatic activity in a cell-free assay but was 10-fold less toxic against human leukaemia cell lines than native RTA. However, Tfn conjugated with cloned RTA (cRTA) is 10- to 20-fold more cytotoxic than Tfn and natural RTA (nRTA). These results suggested that the ability of vesicular stomatitis virus protein G to interact with cell membranes facilitate the translocation of RTA to the cell cytosol.

The mechanisms of facilitating conjugate cytotoxicity by viruses are not completely elucidated due, it has been proposed, to the fusogenic activity by the viral peptide. The acidification of these endosomes by an ATP-dependent proton pump is responsible for initiating the fusion of viral and cellular membranes (Eidelman et al, 1984; Florkiewicz and Rose, 1984). Recently, computer simulations of the structure of KFT25 and calculations of the hydropathicity and of the mean hydrophobic moment of the KFT25 peptide indicated that the peptide is composed of three distinct structural regions separated by Pro residues: an N-terminal hydrophobic α-helix (Lys-Pro region), a central hydrophilic globular structure (His-Pro region) and a slightly hydrophilic C-terminal β-structure (Ser-Pro). The N-terminus can potentially span the first layer of the plasma membrane at pH 7.0 with an emission of 5.3 kcal $mol^{-1}$. This calculation supports the data reported by Schlegel and Wade (1985) that the first six amino acids of KFT25 are haemolytic even at physiological pH, whereas the globular region would be implicated in the pH activation of the haemolytic properties of the entire peptide. The properties of the KFT25 peptide could explain the lower cytotoxic activity of unconjugated cRTA. cRTA might insert into the plasma membrane and may remain entrapped within the lipid layers as a consequence. Unconjugated cRTA has lower translocation potential as compared with nRTA, but once cRTA is vehicled near the membrane by Tfn, cRTA can insert itself into the lipid layer. Upon acidification of the environment, cRTA would increase its N-terminal positive charge because of the His residues. This would lead to a disorganization of the bilayer structure as a result of attracting the negative charges present on the cytosolic surface of the cellular membranes. The disorganization would facilitate the translocation of the cRTA to cell cytosol. A role in the pH activation of the KFT25 properties could also be played by the proline residues.

### Adenovirus

The capacity of adenovirus to disrupt endosomes as part of their entry mechanisms was exploited to enhance the efficiency of gene delivery and cytotoxic conjugates (FitzGerald et al, 1983; Curiel et al, 1991; Wu et al, in preparation). Using electron microscopy, FitzGerald et al (1983) first reported that adenovirus can help release EGF–gold conjugates from the receptosomes into the cytosol. Adenovirus enhanced the toxicity of PE by 100-fold and the cytotoxicity of the IT, consisting of PE–EGF, by 10 000-fold through disruption of receptosomes. Adenovirus infection augmented levels of gene transfer by transferrin–polylysine conjugates in a dose-dependent manner: levels of gene transfer of > 2000-fold were achieved. Adenovirus enhances levels of gene transfer in a variety of targeting cells, including cell lines otherwise refractory to gene transfer by transferrin–polylysine conjugates. The augmentation was based on adenovirus-mediated vesicle disruption, a process independent of viral gene expression. The inhibiting factors for gene transfer were thought to be a consequence of lysosomal targeting of the endosome–internalized conjugate–DNA complexes (Wagner et al, 1994). Agents to inhibit lysosomal enzymes have been used to increase the fraction of DNA that would escape degradation and be expressed within the nucleus. The development of specific mechanisms to effect release from the endosome in combination with gene transfer by the receptor-mediated endocytosis pathway will increase the use of this delivery system by allowing high levels of gene expression in target cells. We also found that adenovirus can increase the cytotoxicity of RTA delivered by bacteriophage MS2 capsids (Wu et al, 1995; in preparation).

### CYTOKINES

O'Boyle et al (1995) reported a conjugate of gelonin and MAb (lym-1) – class II HLA-DR antigen. In vitro cytotoxicity of the conjugate was confirmed by the delivery of MAb lym-1. γ-interferon

© Cancer Research Campaign 1997

(IFN) augmented the antiproliferative effects of lym-1-gelonin conjugate, especially at its low concentration and unconjugated lym-1. Tumour necrosis factor alpha (TNF-α) also enhanced the antiproliferative activity of free lym-1 but did not significantly increase the cytotoxicity of the conjugate. The augmentation of the conjugate may be due to an additive cytotoxicity effect because γ-IFN exhibited some direct cytotoxic activity on Raji lymphoma cells (expressing class II antigen) in the absence of the conjugate. This combined therapy that has an augmented effect may be useful means of treating tumours. It is considered that γ-IFN retards the growth and proliferation of both tumour and normal cells through elongating the cell cycle. γ-IFN works through a signal transduction mechanism by inducing phosphorylation of protein kinase Jak1 and Jak2 as well as γ-IFN receptor. The ability of γ-IFN, and to a lesser degree TNF-α, to significantly enhance the antiproliferative effect of conjugated lym-1 is also remarkable. This suggests some type of synergistic interaction between the cytostatic effects of a growth-regulatory MAb and cytotoxic activity of cytokine, especially γ-IFN which could kill the more slowly growing malignant B cells better than before. The mechanism is probably as a result of activation of kinases and phosphorylated proteins that slow down tumour cell growth and cause the cells to undergo apoptosis. The TNF-α mechanism in vitro is thought to be different from its mechanism in vivo. The haemorrhagic necrosis seen in vivo is the result of its effects on tumour endothelium, generating procoagulant activity and decreased perfusion of the tumour. TNF-α needs to be internalized for its in vitro activity, because chloroquine and colchicine, which disrupt the endocytic process, inhibit its effects. TNF-α participates in the signal transduction pathway by stimulating PKC activity. TNF-α and γ-IFN can also have a synergistic cytotoxic effect on haematopoietic progenitor cells and can induce expression of the apoptosis-associated antigen (FAS or CD95).

Yokota et al (1990) investigated the in vivo effect of recombinant human α-IFN on the anti-tumour activity of ITs containing RTA and anti-human leukaemia MAb SN1 or SN2. SN1 and SN2 are directed towards two unique T-leukaemia-associated antigens, TALLA and GP37 (Yokoda et al, 1993). Nude mice were inoculated with Ichikawa cells and treated for 4 days with ITs plus α-IFN. The results showed 100% inhibition of tumour growth in the treated mice, while similar treatment with each agent alone was only partly effective. The results indicated that IFN potentiates the in vivo anti-tumour activity of the ITs, primarily by host-mediated effector mechanisms but not by direct action of IFN-α on the leukaemia cells. The activation of macrophages by IFN in the tumour-bearing nude mice appears to be the major factor in the potentiation of the in vitro anti-tumour activity of the ITs in this study. IFN exerts a variety of effects on tumour cells at the cellular level. These effects can be divided into two groups, i.e. effects by the direct action of IFN on the tumour cells and the host-mediated effects. IFN potentiated both NK cells and macrophage activity in the tumour-bearing nude mice. Ishikawa leukaemia cells are resistant to NK cell lysis, therefore macrophage activation by IFN appears to be the major factor in the synergistic potentiation of anti-leukaemia activity of the ITs. The results have been supported by Basham et al (1988) and Cameron et al (1988) who demonstrated that the host effector mechanisms were important in potentiating anti-tumour activity of anti-idiotype antibody or interleukin 2 (IL-2) by hIFN-α.

Pearson et al (1993) discovered that hIFN-α can potentiate the activity of an IT against ovarian carcinoma cells and this is dependent on tumour burden. These agents are less effective against large tumour burdens but their beneficial effects re-emerge after cytoreduction by a combination of chemotherapy with conventional chemical drugs (CDM, CDDP). It is important to note that rhIFN-α is species specific (Pearson et al, 1990). These data showing the use of IFN for enhancing ITs provides an idea that the combined therapy of ITs and cytokines may be promising for the treatment of diseases, including tumours and AIDS.

### β-ADRENERGIC BLOCKING AGENTS

IT in vivo activity is often diminished by their ability to gain access to the tumour site in appropriate concentrations. Another problem for ITs is their difficulty in gaining access to tumours via the endothelial and reticuloendothelial barriers. The accessibility of solid tumours by ITs is even worse because of their dense connective tissue and relatively restricted blood supply. Changes in blood flow in and around tumour masses could significantly alter the effectiveness of therapies, and vasoactive drugs can be used to alter the distribution of blood flow between tumour and normal tissues when using ITs. It was found that non-selective and cardioselective β-adrenergic blocking agents could increase threefold tumour-to-blood and tumour-to-liver perfusion of $^{125}$I-labelled MAbs (Smyth et al, 1987). These β-adrenergic blockers increased the anti-tumour efficacy of idarubicin (Ida)–MAb conjugates. These agents in combination with the conjugates produced a smaller mean tumour size and a greater number of regressions than the conjugate-alone groups in the tumour-bearing mice, but prazosin hydrochloride ($\alpha_1$-adrenergic blocking agent) and Cyclosasmol (peripheral vasodilator) could not enhance the tumour perfusion and anti-tumour efficacy of $^{125}$I- or Ida-conjugated MAbs. It was demonstrated that the $\beta_2$-adrenergic blocking effect is not related to this enhancing ability. These results might suggest that β-adrenergic blocking agents are useful in tumour therapy in combination with immunoconjugates.

The studies of $\beta_2$-adrenergic blockers were only limited in the MAb–radioisotope conjugates and its role for enhancing MAb–toxin conjugates has not been reported. Investigation is required on the possible potence of $\beta_2$-adrenergic blockers in targeted therapy.

### CYCLOSPORIN A, DAUNORUBICIN AND RETINOID ACID

### Cyclosporin A

It is difficult to eradicate virtually all neoplastic cells in a heterogeneous population of cells that have the capacity for continuing genetic alteration. Prevention of cancers has been recently emphasized as a more effective means for cancer control. Cyclosporin A is an immunosuppressive cyclic peptide widely used to prevent rejection of allogeneic grafts. Yefernof et al (1992) discussed an issue of using cyclosporin A to potentiate the IT activity of tumour prevention. Cyclosporin A is able to inhibit lymphokine secretion by T cells. The investigators constructed an IT containing deglycosylated RTA (dgA) and a MAb (2F10) against the radiation leukaemia virus (RadLV) envelope glycoprotein (gp70). RadLV is a retrovirus that induces clonal thymic lymphomas in C57BL/6 mice after a latency period of 3–6 months. A pleioclonal population of preleukaemic (PL) cells is not malignant but can progress to

© Cancer Research Campaign 1997

**Table** Enhancement of IT activity by various reagents

| Reagents | Enhancement (fold) | Immunotoxins | Reference |
|---|---|---|---|
| Ammonium chloride | 10–1625 | Anti-T65-RTA, anti-Thy 1.2, anti-T101-RTA | Casellas et al (1984) Casellas et al (1988) |
| β-Glycylphenylnaphthylamide | 10 | Anti-Tfn-PE Epidermal growth factor-PE | Akiyama et al (1985) |
| Methylamine | 50% more inhibition than IT alone | Anti-T101-RTA | Siena et al (1987) Poole and Ohkuma (1981) |
| Amantadine | 98% more inhibition than IT alone | Anti-T101-RTA | Siena et al (1987) |
| Chloroquine | 10–15 | Lutropin-gelonin | Marcil et al (1993) |
| Monensin | 42–420 | RTA-IT | Singh et al (1994) |
| Perhexiline | 10–2000 | Anti-CD5-RTA, T101, T101 $F(ab)_2$ | Gaffrezou et al (1990) |
| Verapamil | 2–60 | RTA-anti-Tfn receptor HB21-PE | Pirker et al (1989) |
| Viral peptide | 10–20 | Tfn-RTA-KFT viral peptide | Chignola et al (1995) |
| Adenoviruses | ~ 10 000 | PE-EGF | FitzGerald et al (1983) |
| | > 2000 gene transfer efficiency | Tfn-polylysine-DNA complex | Curiel et al (1991) |
| Interferons | 100% inhibition of tumour growth | Anti-T leukaemia Ab-RTA | Yokota et al (1990) |
| β-Adrenergic blockers | threefold tumour to blood perfusion | I-labelled MAb conjugates | Smyth et al (1987) |
| Cyclosporin A | 100 | Anti-CD5-RTA | Jeffrezou et al (1994) |
| | Eradication of RadLV-infected cells | Anti-RadLV-dgRTA | Yefernof et al (1992) |
| Daunorubicin | 80% inhibition of tumour growth | Anti-T leukaemia Ab-RTA | Yokota et al (1990) |
| Retinoic acid | > 10 000 | Anti-Tfn Ab 454A12-RTA | Wu et al (1994) |
| Ricin B-chain | From non-effect to effect | RTA-IT | McIntosh et al (1983) |
| B-chain IT (same Ab in RTA-IT) | Several folds | RTA-IT to neoplastic B cells | Vitetta et al (1983) |
| Piggyback B-chain IT | Several folds | RTA-IT to neoplastic B cells | Vitetta et al (1984) |
| KDEL peptide | Markedly | PE-ITs | Chaudhary et al (1990) |
| KDEL peptide | Significantly | RTA | Wales et al (1993) |

lymphoma. Long-term existence of the PL cells after inoculating thymus has been attributed to the ability of the virus to induce both interleukin 4 (IL-4) and IL-4 receptor expression. The IT 2F10-dgA eradicated the host majority of RadLV-infected cells. This treatment delayed the premalignant process as the few surviving PL cells are still capable of progressing into malignant lymphoma. With the administration of the IT plus cyclosporin, the escaping PL cells were eliminated through blocking the IL-4 secretion. The mechanisms are not clear, but one of the profound biological activities is the inhibition of lymphokine production and secretion in activated T cells. This terminates malignant development.

Jaffrezou et al (1994) evaluated the ability of cyclosporin A and its non-immunosuppressive analogue, SDZ PSC 833, to enhance anti-CD5 RTA ITs in vitro. At 4 μM, both reagents increased the cytotoxicity of the anti-CD5 IT on the human lymphoblastic T-cell line CEM by 100 times. These reagents could also increase anti-CD5 $F(ab')_2$ RTA IT (a more potent IT) by 8–9 times. They did not affect the rate of RTA-IT binding, internalization, intracellular trafficking or degradation. It was found that the internalized anti-CD5 IT were intact, which suggests that the enhancers may act only on a small population of RTA IT that escapes present investigational techniques. The study of the in vivo toxicity of cyclosporin A demonstrated that a concentration of 4 μM in the body did not result in long-term immunosuppressive consequences. The main side-effect is capillary-leak syndrome (possibly related to immunosuppressive activity) which could be treated by gluco-conicoids. This indicates that the non-immunosuppressive analogue SDZ PSC 833 is more appropriate in the clinical application, because SDZ PSC 833 is more effective in enhancing the activity of IIs.

### Daunorubicin

Daunorubicin is an antibiotic of the rhodomycin group and is being widely used for treating human leukaemias; daunorubicin inhibits DNA and RNA synthesis in the cell. Daunorubicin kills target cells by a different machanism from cytotoxic ITs. The inhibition of nucleotide synthesis can lead to the inhibition of protein synthesis as polynucleotides are necessary for protein synthesis. It was shown that daunorubicin could facilitate the activity of the ITs against human T-leukaemia-associated antigens (Yokota et al, 1990). In nude mice T-leukaemia models, daunorubicin plus ITs suppressed the tumour growth up to 80%, which is similar to ITs plus IFN, however ITs with IFN plus daunorubicin could result in 100% suppression of tumour growth. Thus the combined action of daunorubicin and ITs could achieve additive cytotoxicity through multiple mechanisms. Griffin et al (1989) also found that doxorubicin, another chemotherapeutic agent, potentiated in vivo anti-tumour activity of an anti-human transferrin receptor IT.

### Retinoic acid

Retinoic acid can selectively increase the potency of some ITs (Wu et al, 1994). Retinoic acid increases the activity of ITs with RTA but does not increase the activity of ITs from diphtheria toxin and PE. BFA can block retinoic acid-mediated IT potentiation but not

© Cancer Research Campaign 1997

monensin-mediated IT potentiation. This demonstrated that retinoic acid possesses some characteristics of IT potentiation similar to monensin, but some different from monensin. This indicates that retinoic acid might be a new reagent to manipulate the Golgi apparatus, but in vivo experiments are required to analyse the ability to enhance the efficacy of ITs.

### OTHERS

### Introduction of KDEL peptide

The sequence Arg-Glu-Asp-Leu-Lys (REDLK) at the carboxyl terminus of PE is known to be important for its cytotoxicity, though it is not required for the binding and enzymic process. The REDLK is strikingly similar to the endoplasmic reticulum retrieval sequence KDEL (Chaudhary et al, 1990; Seetharam et al, 1991). During the intoxication process, PE is cleaved by an intracellular protease between amino acids 279 and 280 to generate an Mr 37 000 carboxyl terminal fragment that contains the ADP-ribosylation activity. This fragment appears to be directed to the endoplasmic reticulum by the REDLK sequence at its carboxyl terminus, from which compartment it translocates to the cytosol. If REDLK is replaced by KDEL, the cytotoxic activity of PE can be increased. PE contains a KDEL-like C-terminal sequence that can bind the KDEL-receptor in the Golgi that is proposed to carry PE out of the Golgi to the endoplasmic reticulum for efficient transport to the cytosol. A number of fusion proteins that contain PE domain III with a KDEL peptide linked to various ligands, including transforming growth factor α, EGF, Tfn, EGF-like domain of heregulins, IL-2, IL-4, IL-6, etc., have been studied (Pai and Pastan, 1993; Gottstein et al, 1994; Thrush et al, 1996). The cytotoxicity of all the fusion proteins are increased when the carboxyl terminus is attached with a KDEL. Lord and his colleagues (Wales et al, 1993) developed a series of fusion proteins by replacing the KEDLP of ricin A-chain with a KDEL sequence. A considerable increase in the cytotoxicity was also achieved. The results in our laboratory also showed that RTA-KDEL conjugates carried with MS2 bacteriophage coat protein were more cytotoxic to the target cells (Wu et al, unpublished). Thus the KDEL-derivatized conjugates hold stronger cytotoxicity, and these ITs may be better agents than the conjugates containing a native toxin for the therapeutic application.

### Synergy of ricin B-chain and its ITs

We discussed in the opening section the role of ricin B-chain, which is important for ITs although the B-chain is not required to exert the protein synthesis inhibition. B-chain aids the translocation of the A-chain and probably protects the A-chain from degradation. Therefore several strategies have been tried to increase the RTA-IT activity with the B-chain. A direct approach is to add free ricin B-chain after target cells have bound to RTA-ITs, as free B-chain can bind to the A-chain of ITs more readily than they bind to the galactose-containing glycoproteins on the cell surface. It was found that the B-chain potentiated the toxicity of RTA-ITs in vitro when the concentration of extraneous glycoproteins was low (McIntosh et al, 1983). Use of the B-chain in vivo seems unlikely because of the much higher concentration of glycoproteins and cells bearing these glycoproteins in the blood. Thus free B-chain will be diminished before reaching the target cells of ITs.

Vitetta et al (1983) pioneered the use of a B-chain IT to improve an IT with RTA. The idea was that if both the ITs were bound to the same cell, they would be endocytosed within the same vesicle and cleaved from their IT, allowing the B-chain to perform translocation of the A-chain. In vitro assays confirmed that the B-chain IT potentiated the A-chain IT. Again by Vitetta's group, the approach was extended by generating a B-chain IT in which the antibody is reactive to the antibody of the RTA-IT, namely 'piggyback' (Vitetta et al, 1984). This experiment in the use of the B-chain IT for enhancement was described as being successful. It is necessary to stress that these strategies require highly purified ITs by affinity chromatography or the free A- and B-chain will reform, resulting in non-specific strong toxicity. There were no further developments in the investigation, thus there may be problems in the pharmacology or methodology. The reagents for enhancing IT activity are summarized in the table.

### CONCLUSIONS

Treatment of tumours and some other refractory diseases are long-term issues in which considerable progress has been achieved over the last 20 years. However, the therapeutic strategies that are being developed are not panacea and usually enjoy only partial success. The treatment of tumours with ITs in combination with some other drugs or chemical reagents, such as cytokines, amines, calcium channel antagonists and carboxylic ionophores, can markedly potentiate anti-tumour effect.

We propose here that the treatment of refractory diseases by combined therapy may yet prove a means of controlling diseases. The use of ITs together with enhancing reagents has potential, and it is therefore worthwhile investigating its application.

### ACKNOWLEDGEMENT

I would like to thank A Parrott and others for English corrections.

### REFERENCES

Akiyama S, Gottesman MM, Hanover JA, FitzGerald D, Willingham MC and Pastan I (1984) Verapamil enhances the toxicity of conjugates of epidermal growth factor with *pseudomonas* exotoxin and antitransferrin receptor with *pseudomonas* exotoxin. *J Cell Physiol* **120**: 271–279

Akiyama S, Seth P, Pirker R, FitzGerald D, Gottesman MM and Pastan I (1985) Potentiation of cytotoxic activity of immunotoxins on cultured human cells. *Cancer Res* **45**: 1005–1007

Basham TY, Race ER, Campbell MJ, Reid TR, Levy R and Merigan TC (1988) Synergistic antitumor activity with IFN and monoclonal antiidiotype for murine B cell lymphoma: mechanism of action. *J Immunol* **141**: 2855–2860

Cameron RB, McIntosh JK and Rosenberg SA (1988) Synergistic antitumor effects of combination immunotherapy with recombinant interleukin-2 and a recombinant hybrid α-interferon in the treatment of established murine hepatic metastases. *Cancer Res* **48**: 5810–5817

Casellas P, Bourrie BJP, Gros P and Jansen FK (1984) Kinetics of cytotoxicity induced by immunotoxins, enhancement by lysosomotropic amines and carboxylic ionophores. *J Biol Chem* **259**: 9359–9364

Casellas P, Ravel S, Bourrie BJP, Derocq JM, Jansen FK, Laurent G and Gros P (1988) T-lymphocyte killing by T101-ricin A chain immunotoxin: pH-dependent potentiation with lysosomotropic amines. *Blood* **72**: 1197–1202

Chaudhary VK, Jinno Y, FitzGerald D and Pastan I (1990) *Pseudomonas* exotoxin contains a specific sequence at the carboxyl terminus that is required for cytotoxicity. *Proc Natl Acad Sci USA* **87**: 308–312

Chignola R, Anselmi C, Serra MD, Franceschi A, Fracasso G, Pasti M, Chiesa E,

© Cancer Research Campaign 1997

Lord JM, Tridente G and Colombatti M (1995) Self-potentiation of ligand-toxin conjugates containing ricin A chain fused with viral structures. *J Biol Chem* **270**: 23345–23351

Colombatti M, Dell'Arciprete L, Chignola R and Tridente G (1990) Carrier protein-monensin conjugates: enhancement of immunotoxin cytotoxicity and potential in tumor treatment. *Cancer Res* **50**: 13385–13391

Curiel DT, Agarwal S, Wagner E and Cotten M (1991) Adenovirus enhancement of transferrin-polylysine-mediated gene delivery. *Proc Natl Acad Sci USA* **88**: 8850–8854

Eidelman O, Schlegel R, Tralka T and Blumenthal R (1984) pH-dependent fusion induced by vesicular stomatitis virus glycoprotein reconstitutes into phospholipid vesicles. *J Biol Chem* **259**: 4622–2628

Endo Y, Mitsui K, Motizuki M and Tsurugi K (1987) The mechanism of action of ricin and related toxic rectins on eukaryotic ribosomes. *J Biol Chem* **262**: 5908–5912

FitzGerald D, Padmanabhan R, Pastan I and Willingham MC (1983) Adenovirus-induced release of epidermal growth factor and Pseudomonas toxin into the cytosol of KB cells during receptor-mediated endocytosis. *Cell* **32**: 607–617

Florkiewicz R and Rose J (1984) A cell line expressing vesicular stomatitis virus glycoprotein fuses at low pH. *Science* **225**: 721–723

Gottstein C, Winkler U, Bohlen H, Diehl V and Engert A (1994) Immunotoxins: is there a clinical value? *Ann Oncol* **5** (suppl. 1): s97–s103

Griffin T and Raso V (1991) Monersin in lipid emulsion for the potentiation of ricin-A chain immunotoxins. *Cancer Res* **51**: 4316–4322

Griffin T, Rybak ME, Recht L, Singh M, Salimi A and Raso V (1993) Potentiation of antitumor immunotoxins by liposomal monensin. *J Natl Cancer Inst* **85**: 292–298

Jaffrezou JP, Levade T, Kuhlein E, Thurneyssen O, Chiron M, Grandjeas H, Carriere D and Laurent G (1990) Enhancement of ricin A chain immunotoxin activity by perhexiline on established and fresh leukemia cells. *Cancer Res* **50**: 5558–5566

Jaffrezou JP, Sikic BI and Laurent G (1994) Cyclosporine A and cyclosporine SDZ PSC 833 enhance anti-CD5 ricin A-chain immunotoxins in human leukemic T-cells. *Blood* **83**: 482–489

Jansen FK, Jansen A, Dercoq JM, Carriere D, Carayon P, Veas F and Jaffrezou JP (1992) Golgi vacuolization and immunotoxin enhancement by monensin and perhexiline depend on a serum protein. Implications for intracellular trafficking. *J Biol Chem* **267**: 12577–12682

Lendaro E, Ippoliti R, Belleli A, Brunori M, Evangelista V, Guidarini D and Benedetti PA (1994) Intracellular dynamics of ricin followed by fluorescence microscopy on living cells reveals a rapid accumulation of the dimeric toxin in the Golgi apparatus. *FEBS Lett* **344**: 99–104

Marcil J, Ravindranath N and Sairam MR (1993) Cytotoxic activity of lutropin-gelonin conjugate in mouse leydig tumor-cells – potentiation of the hormonotoxin activity by different drugs. *Mol Cell Endocrinol* **92**: 83–90

McIntosh DP, Edwards DC, Cumber AJ, Parnell GD, Dean CJ, Ross WCJ and Forrester JA (1983) Ricin B chain converts a non-cytotoxic antibody-ricin A chain conjugate into a potent and specific cytotoxic agent. *FEBS Lett* **164**: 17–20

Mezei M and Nugeni FJ (1984) Method of encapsulating biologically active materials in multilamellar lipid vesicles. US Patent **4**: 485054

Mollenhauer H, Morre DJ and Rowe LD (1990) Alteration of intracellular traffic by monensin, mechanism, specificity and relationship to toxicity. *Biochimica et Biophysica Acta* **1031**: 225–246

O'Boyle KP, Colletti D, Mazurek C, Wang Y, Ray SK, Diamond B, Rosenblum MG, Epstein AL, Shochat D, Dutcher JP, Wiernik PH and Klein RS (1995) Potentiation of antiproliferative effects of monoclonal antibody LYM-1 and immunoconjugate LYM-1-gelonin on human Burkitts-lymphoma cells with Gamma-interferon and tumor-necrosis-factor. *J Immunother* **18**: 221–230

Pai LH and Pastan I (1993) Immunotoxin therapy for cancer. *JAMA* **269**: 78–81

Pearson JW, Hedrick E, Fogler WE, Bull RL, Ferris DK, Riggs CW, Wiltrout RH, Sivam G, Morgan AC, Groves ES and Longo DL (1990) Enhanced therapeutic efficacy against an ovarian tumor xenograft of immunotoxins used in conjunction with recombinant α-interferon. *Cancer Res* **50**: 6379–6388

Pearson JW, Fogler WE, Volker K, Riggs CW, Gruys E, Groves ES, Wiltrout RH and Longo DL (1993) Restoration of interferon-alpha potentiation of a recombinant ricin-A chain immunotoxin following cytoreduction of xenografts of advanced ovarian-tumors. *J Natl Cancer Inst* **85**: 907–912

Pirker R, FitzGerald DJP, Raschack M, Frank Z, Willingham MC and Pastan I (1989) Enhancement of the activity of immunotoxins by analogues of verapamil. *Cancer Res* **49**: 4791–4795

Poole B and Ohkuma S (1981) Effect of weak bases on the intralysosomal pH in mouse peritoneal macrophages. *J Cell Biol* **90**: 665–669

Roth JA, Ames RS, Fry K, Lee HM and Scannon PJ (1988) Mediation of reduction of spontaneous and experimental pulmonary metastases by ricin A chain immunotoxin 45-2D9-RTA with potentiation by systemic monensin in mice. *Cancer Res* **48**: 3496–3501

Schlege R and Wade M (1985) Biologically active peptides of the vesicular stomititis virus glycoprotein. *J Virol* **53**: 319–323

Seetharam S, Chaudhary VK, FitzGerald D and Pastan I (1991) Increased cytotoxic activity of *pseudomonas* exotoxin and two chimeric toxins ending in KDEL. *J Biol Chem* **266**: 17376–17381

Siena S, Villa S, Bregni M, Bonnadonna G and Gianni AM (1987) Amantadine potentiates T lymphocyte killing by an anti-pan-T cell (CD4) ricin A-chain immunotoxin. *Blood* **69**: 345–348

Singh M, Griffin T, Salimi A, Micetich RG and Atwal H (1994) Potentiation of ricin-A immunotoxin by monoclonal-antibody targeted monensin containing small unilamellar vesicles. *Cancer Lett* **84**: 15–21

Smyth MJ, Pietersz GA and Mckenzie IFC (1987) Use of vasoactive agents to increase tumor perfusion and the antitumor efficacy of drug-monoclonal antibody conjugates. *J Natl Cancer Inst* **79**: 1367–1373

Thrush GP, Lark LR, Clinchy BC and Vitetta ES (1996) Immunotoxins: an update. *Annu Review Immunol* **14**: 49–71

Uhlin-hansen L and Yanagishita M (1995) Brefeldin A inhibits the endocytosis of plasma-membrane-associated heparan sulphate proteoglycans of cultured rat ovarian granulosa cells. *Biochem J* **310**: 271–278

Vallera DA (1994) Immunotoxins: will their clinical promise be fulfilled? *Blood* **83**: 309–317

Vitetta ES, Cushley W and Uhr JW (1983) Synergy of ricin A chain-containing immunotoxins and ricin B chain-containing immunotoxins in the in vitro killing of neoplastic human B cells. *Proc Natl Acad Sci USA* **80**: 6332–6335

Vitetta ES, Fulton RJ and Uhr JW (1984) The cytotoxicity of cell-reactive immunotoxin containing ricin A chain is potentiated with an anti-immunotoxin containing ricin B chain. *J Exp Med* **160**: 341–346

Wagner E, Curiel DT and Cotten M (1994) Delivery of drugs, proteins and genes into cells using transferrin as a ligand for receptor-mediated endocytosis. *Adv Drug Delivery Review* **14**: 113–135

Wales R, Roberts LM and Lord JM (1993) Addition of an endoplasmic reticulum retrieval sequence to ricin A chain significantly increases its cytotoxicity to mammalian cells. *J Biol Chem* **268**: 23986–23990

Wu M, Tang SL, Zang RJ and Yu H (1990) Selective killing of tumor cells by an immunotoxin composed of ricin and monoclonal antibody against Ia antigen. *Int J Immunopharmacol* **12**: 235–239

Wu M, Miyazawa M and Nose M (1993) Immunotoxins as a new reagent for treatment of viral infection and autoimmune diseases. *Drug Delivery System* **8**: 5–10

Wu M, Brown WL and Stockley PG (1995) Cell-specific delivery of bacteriophage-encapsidated ricin A chain. *Bioconjugate Chem* **6**: 587–595

Wu YN, Gadina M, Taocheng JH and Youle RJ (1994) Retinoic acid disrupts the Golgi-apparatus and increases the cytosolic routing of specific protein toxins. *J Cell Biol* **125**: 743–753

Yefenof E, Abboud G, Epszteyn S and Vitetta ES (1992) Treatment of premalignancy: prevention of lymphoma in radiation leukemia virus-inoculated mice by cyclosporin A and immunotoxin. *Proc Natl Sci Acad USA* **89**: 728–732

Yokota S, Hara H, Guo Y and Seon BK (1990) Synergistic potentiation of *in vivo* antitumor activity of anti-human T-leukemia immunotoxins by recombinant α-interferon and daunorubicin. *Cancer Res* **50**: 32–37

Yokota S, Okazaki M, Yoshida M and Seon BK (1993) Biodistribution and *in vivo* antitumor efficacy of the systemically administrated anti-tumor T-leukemia immunotoxins and potentiation of their efficacy by α-interferon. *Leukemia Res* **17**: 69–79

© Cancer Research Campaign 1997